# Construction and validation of nursing diagnoses for premature newborns

**DOI:** 10.1590/1980-220X-REEUSP-2023-0167en

**Published:** 2023-11-20

**Authors:** Danielle Lemos Querido, Marialda Moreira Christoffel, Viviane Saraiva de Almeida, Ana Paula Vieira dos Santos Esteves, Harlon França de Menezes, Halene Cristina Dias de Armada e Silva, Alessandra Conceição Leite Funchal Camacho

**Affiliations:** 1 Universidade Federal do Rio de Janeiro, Maternidade Escola, Rio de Janeiro, RJ, Brazil.; 2 Universidade Federal do Rio de Janeiro, Escola de Enfermagem Anna Nery, Rio de Janeiro, RJ, Brazil.; 3 Hospital Pró-Cardíaco, Rio de Janeiro, RJ, Brazil.; 4 Secretaria Municipal de Saúde do Rio de Janeiro, Rio de Janeiro, RJ, Brazil.; 5 Universidade Federal Fluminense, Escola de Enfermagem Aurora de Afonso Costa, Niterói, RJ, Brazil.

**Keywords:** Classification, Standardized Nursing Terminology, Nursing Diagnosis, Infant, Newborn, Intensive Care Units, Neonatal, Clasificación, Terminología Normalizada de Enfermería, Diagnóstico de Enfermería, Recién Nacido, Unidades de Cuidados Intensivos Neonatal, Classificação, Terminologia Padronizada em Enfermagem, Diagnóstico de Enfermagem, Recém-Nascido, Unidades de Terapia Intensiva Neonatal

## Abstract

**Objective:**

To build and validate nursing diagnoses based on the International Classification of Nursing Practice (ICNP^®^) for premature newborns admitted to the Neonatal Intensive Care Unit.

**Method:**

Methodological study based on the Brazilian method for developing subsets: use of specialized nursing language terms, construction of diagnostic statements and content validation of the statements by 40 specialist nurses. Those with a Content Validity Index (CVI) ≥ 0.80, organized according to Wanda Horta’s basic human needs theory, were considered valid.

**Results:**

146 nursing diagnosis statements were constructed and 145 (93.3%) diagnoses were validated, with a predominance of the human need for cutaneous-mucosal integrity.

**Conclusion:**

The specificity of neonatal care is evident when these diagnoses are presented and validated in order to support nurses in their clinical reasoning and decision-making.

## RESUMO

**Objetivo:**

Construir e validar diagnósticos de enfermagem fundamentados na Classificação Internacional da Prática de Enfermagem (CIPE^^®^^) para recém-nascidos prematuros internados em Unidade de Terapia Intensiva Neonatal.

**Método:**

Estudo metodológico sustentado pelo método brasileiro para desenvolvimento de subconjuntos: utilização de termos da linguagem especializada de enfermagem, construção dos enunciados de diagnósticos e validação de conteúdo dos enunciados por 40 enfermeiros especialistas. Foram considerados válidos aqueles com Índice de Validade de Conteúdo (IVC) ≥ 0.80, organizados conforme à teoria das necessidades humanas básicas de Wanda Horta.

**Resultados:**

Foram construídos 146 enunciados de diagnósticos de enfermagem, e ao final foram validados 145 (93,3%) diagnósticos, com predominância na necessidade humana de integridade cutâneo-mucosa.

**Conclusão:**

A especificidade do cuidado neonatal fica evidente a partir do momento em que se apresentam tais diagnósticos, e que são validados com o objetivo de subsidiar o enfermeiro no raciocínio clínico e na tomada de decisão.

## RESUMEN

**Objetivo:**

Construir y validar diagnósticos de enfermería basados en la Clasificación Internacional de la Práctica de Enfermería (CIPE^®^) para recién nacidos prematuros ingresados en la Unidad de Cuidados Intensivos Neonatales.

**Método:**

Estudio metodológico basado en el método brasileño de elaboración de subconjuntos: utilización de términos del lenguaje enfermero especializado, construcción de enunciados diagnósticos y validación de contenido de los enunciados por 40 enfermeros especialistas. Se consideraron válidos aquellos con Índice de Validez de Contenido (IVC) ≥ 0,80, organizados según las necesidades humanas básicas de Wanda Horta.

**Resultados:**

Se construyeron 146 enunciados de diagnóstico de enfermería y se validaron 145 (93,3%) diagnósticos, con predominio de la necesidad humana de integridad cutáneo-mucosa.

**Conclusión:**

La especificidad de los cuidados neonatales es evidente desde el momento en que estos diagnósticos son presentados y validados con el objetivo de subsidiar a las enfermeras en su razonamiento clínico y toma de decisiones.

## INTRODUCTION

According to the World Health Organization (WHO), the rate of prematurity has been increasing in recent decades, reaching 14.8 million premature births worldwide in 2014, which represented 10.6% of all births. Notwithstanding this, Brazil showed a downward trend in these rates between 2012 and 2019, ranging from 10.87% to 9.95%. However, the country still has a high proportion of prematurity compared to European countries (8.7%)^(1,2)^.

Premature birth most often involves hospitalization in the neonatal intensive care unit (NICU) where specific care for this population is concentrated, developed by a multi-professional team. In this scenario, nurses provide comprehensive and individualized care based on the operationalization of the nursing process, as recommended by Resolution 358 of the Federal Nursing Council, and documented through the use of classification systems^(3)^.

Regarding these issues, there is the International Classification for Nursing Practice (ICNP^®^) with statements of nursing diagnoses (ND), nursing interventions (NI) and nursing outcomes (NO), standing out as a tool to support effective clinical decision-making and the description of professional practice in an organized manner. The standardized language of the ICNP^®^ can be used in different contexts in the neonatal and pediatric areas to describe nursing care^(4)^.

Among these phenomena, ND is considered to be a term referring to a finding, event, situation or other health aspect resulting from data collection, i.e. something that merited the nurse’s attention and was assessed as a real or potential problem and should be identified and recorded during the nurse’s care using standardized terminology^(3,4)^.

The literature shows that the use of standardized nursing terminology leads to an improvement in the quality of care and patient safety, as well as the implementation of the possibility of conducting research, as demonstrated by a review study that identified 33 publications related to the pediatric setting and 11 related to the neonatal setting, concluding that ICNP^®^ can be used to describe the care provided in different age groups, in hospital or out-of-hospital contexts and in cases of specific clinical situations, adapting to different conceptual theoretical models^(5,6)^.

Building nursing diagnoses and their inclusion in information systems can facilitate clinical decision-making, impacting on the quality of care and strengthening nursing as a science^(7)^. However, there is a gap when it comes to publications aimed at the population of premature newborns admitted to the NICU, leaving this clientele lacking a specific subset for their care. Furthermore, when we find publications associating ICNP^®^ with neonatal care, sets of ND/NI and even interventions exclusive to one health context are described^(8,9)^. In this sense, this study is innovative in that it aims to list the ND/NI for the clientele of premature newborns admitted to the NICU, regardless of their underlying disease or admission diagnosis.

In addition, the adoption of a theoretical model to construct the diagnoses enhances nursing praxis, which in this case was based on Wanda Horta’s Basic Human Needs Theory. This theory allows for a holistic view of the newborn, since care for this population is not just related to a disease as such, but to a set of risk factors and real and potential needs, in an individual and specific way, contemplating human needs in their various dimensions, with a view to maintaining health and preventing diseases and complications^(10)^.

Against this backdrop, the aim of this study was to construct and validate nursing diagnoses based on the ICNP^®^ for premature newborns admitted to the Neonatal Intensive Care Unit.

## METHOD

### Study Design

Methodological study, supported by the Brazilian method for developing terminological subsets, carried out between August 2020 and January 2021^(11)^. Based on this, the following steps were carried out: 1) Identification of specialized nursing language terms; 2) Cross-mapping between the terms identified and the ICNP^®^; 3) Construction of nursing diagnostic statements; and, 4) Content validation of nursing diagnostic statements by specialist nurses.

### Data Collection

In the first stage, a previously prepared terminology was used. This was a survey of 2,520 nursing records (nursing notes taken by technicians and nurses on duty) from 70 medical records of premature newborns admitted to the NICU of a public maternity hospital in Rio de Janeiro (RJ) and 70 publications found in national and international databases. The sample included the medical records of newborns admitted to the NICU according to the following inclusion criteria: gestational age at admission ≤ 36 weeks; having at least one nursing record during admission. Newborns who had been readmitted were excluded, so that they wouldn’t be counted more than once; and those who had been transferred to another unit, due to the unavailability of medical records. This investigation collected 418 terms that constituted specialized nursing language documentation for neonatal clients and was used as a starting point for the development of NDs, which is the subject of this publication^(7)^.

This study also applied the International Standard Organization (ISO) 12300: 2016, which addresses standards for mapping between terminology systems, providing subsidies for the creation of clinical terminologies or subsets for specific use. The 418 terms found were classified according to equivalence and cardinality. To reduce bias in this process, cross-mapping was carried out by three researchers.

Between March and December 2020, with these specific terms, nursing diagnoses were developed based on the guidelines of the International Council of Nurses (ICN) and ISO Standard 18.104:2014 - Health informatics: categorical structures for representing nursing diagnoses and nursing actions in terminology systems, in which a diagnosis can be expressed as a judgment about a focus or as the expression of a single clinical finding that represents an altered state, characterizing the second stage of the study^(12)^. The diagnostic statements constructed were entered into a *Microsoft Excel for Windows* spreadsheet, version 2013, and then compared through cross-mapping with the precombined diagnoses of ICNP^®^, version 2019/2020.

The specific wording of the diagnoses was adjusted in terms of spelling, based on the wording contained in the ICNP^®^ and distributed in the psychobiological human needs theory proposed by Wanda Horta, which are appropriate for the study clientele: oxygenation, hydration, nutrition, elimination, sleep and rest, exercise and physical activities, sexuality, shelter, body mechanics, motility, body care, mucosal skin integrity, physical integrity, regulation (thermal, electrolytic, immunological, vascular), locomotion, perception (painful, auditory, visual), environment, therapeutics^(13)^.

The operational definitions of each nursing diagnosis statement were then developed based on a review of the literature in the area (manuals, protocols, publications, books and dictionaries), taking into account the specific nature of neonatal care, which establishes unique characteristics for these statements. These stages took place from August to December 2020.

In order to update the set of proposed diagnostic statements, they were compared to the *Systematized Nomenclature of Medicine International - Clinical Terms* (SNOMED CT). In 2020, the ICN announced *SNOMED International* and the partnership with ICNP^®(14)^. It should be noted that SNOMED CT does not have a translation into Brazilian Portuguese, and it was necessary to have it analyzed by a language professional in order to compare the linguistic equivalence of the statements prepared with those of SNOMED CT, which are presented in the results.

### Selection of Experts

For the third stage of the study, 10 specialist nurses from different regions of Brazil were selected via the Lattes Platform. After this selection, the snowball technique was adopted, where initial participants indicated new participants. The requirement were: to be nurses from the teaching or care areas, with a minimum of five years’ experience in neonatology or nurses who use ICNP^®^ terminology in their daily lives and with a minimum specialist qualification. A total of 78 nurses were selected and, after being invited to take part in the research via email, 40 nurses accepted. They were sent the Informed Consent Form (ICF) in which they had to register their acceptance and follow up the survey by answering the questions on the form.

The statements were divided into three different electronic forms, with an equivalent number of NDs (form 1 consisting of 48 NDs sent to 24 specialists, form 2 consisting of 53 NDs sent to 23 specialists and form 3 consisting of 45 NDs sent to 31 specialists). Since the number of statements of nursing diagnoses was too large to be validated by a single specialist, we opted for this strategy in order to reduce the time taken for validation and increase adherence to the survey and the satisfactory return of the forms.

The first item on the form was aimed at drawing up a professional profile of the specialist nurses. In the second item, an ND was presented in individual statements, followed by the human need in which it was inserted and its operational definition. In this way, the specialist had to give their opinion on the degree of agreement with the statements according to the criteria of relevance and clarity and this degree of agreement. In addition, the form had a space for the experts to describe in detail the suggestions and justifications for their answers.

### Data Analysis and Treatment

To analyze the data, the study used the Content Validity Index (CVI) to measure the percentage of experts who agreed with the aspects presented; statements with a CVI ≥ 0.80 were validated.

The index was calculated using a four-point Likert scale ranging from one (strongly disagree) to four (strongly agree). Items that received a score of “1” (strongly disagree) or “2” (disagree) were reviewed. The index was calculated from the sum of each judge’s “3” (agree) and “4” (totally agree) responses on each item. This sum was then divided by the total number of responses and presented in tables and figures using descriptive statistics^(15)^.

With regard to the evaluation criteria for each item, the statement was maintained whenever relevance and clarity were above 80%. In cases where the evaluation was lower, the statement was adjusted and submitted to a new evaluation.

### Ethical Aspects

This study complied with the ethical requirements of Resolution 466/12 of the National Health Council, maintaining the anonymity of the participants whose medical records were researched and of the experts who took part in the validation process, and obtained a favorable opinion from the proposing institution (No. 2.618.413) and co-participant (2.684.047), both approved in 2018.

## RESULTS

The statements of nursing diagnoses were constructed on the basis of the available literature in the field, following the methodology proposed in the study, and in the end 146 statements were counted. After cross-mapping, it was observed that 54 ND/NI were present in the ICNP^®^ terminology and 92 were new, constructed during the research.

Following the reasoning of dividing the content to be validated into three separate forms, 51.3% of the forms sent were returned. With regard to the profile of these specialists, 22.5% (9) had a doctorate, 42.5% (17) a master’s degree and 35% (14) a specialization degree. As for length of training, the majority (57.5%, 23) had more than 15 years of training. This same figure was repeated in relation to length of professional experience, where 17.5% (7) specialists worked alone in the teaching area, 45% (18) worked alone in the care area and 37.5% worked in both areas. Through all their expertise, the specialist nurses were able to validate the content regarding the relevance and clarity of the ND/NI, as shown in the charts 1 and 2.

**Chart 1:** Validated nursing diagnosis statements for premature newborns admitted to the Intensive Care Unit, according to psychobiological human needs – Rio de Janeiro, RJ, Brazil, 2023.

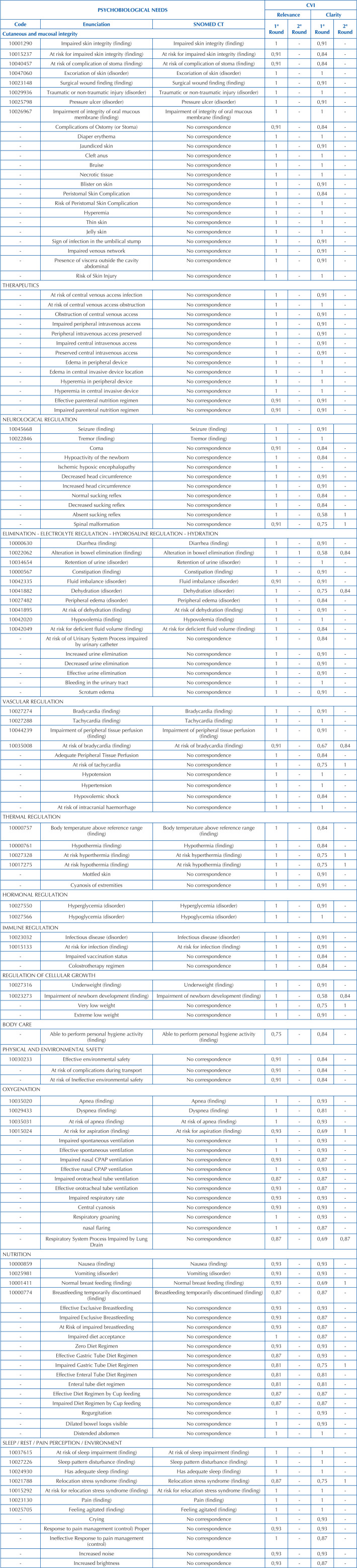

**Chart 2:** Validated nursing diagnosis statements for premature newborns admitted to the Intensive Care Unit, according to psychosocial needs – Rio de Janeiro, RJ, Brazil, 2023.

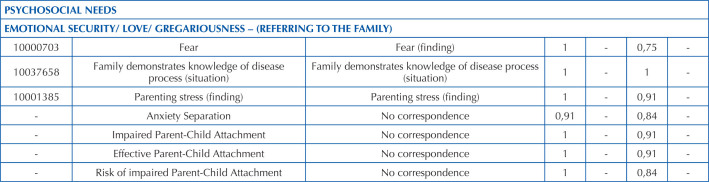

Decision was made regarding the exclusion of ND/NI that had a CVI ≤ 0.80 in relation to relevance, on the understanding that their inclusion in the subset would not be important in the experts’ assessment. Of the 146 statements constructed, only the statement “Ability to perform hygiene absent” did not achieve satisfactory CVI in the relevance item and was excluded from the results.

There were some ND/NI that had a satisfactory CVI in terms of relevance, but ≤ 0.80 in terms of clarity. For these, adjustments were made based on the experts’ suggestions and submitted to a second round of validation in relation to the clarity of the operational definition. They were: “Suction Reflex, Absent”; “Spinal Malformation”; “Dehydration”; “Intestinal Elimination, Impaired”; “Risk of Bradycardia”; “Risk of Tachycardia”; “Risk of Hyperthermia”; “Risk of Hypothermia”; “Newborn Development, Impaired”; “Very Low Weight”; “Fear”; “Risk of Aspiration”; “Respiratory System Process, Impaired with Lung Drain”; “Breastfeeding, Effective”; “Gastric Tube Diet, Impaired”; “Stress from Change (or Transfer) of Environment”.

After these adjustments, they all reached a CVI ≥ 0.80, so of the initial total of 146 ND/NI, 145 were validated and 1 was excluded as not relevant.

## DISCUSSION

Preterm birth is a key risk factor for children development and the severity of birth weight, combined with other issues, has a significant impact on the different dimensions of development. Neurodevelopmental delays and other sequelae have an inversely proportional risk to the baby’s birth weight, appearing in greater numbers in extreme low birth weight populations when compared to groups of babies born at term^(16)^.

Taking this into account, the diagnostic statements validated here are based on affected needs that have repercussions on the integrality of the newborn, and which deserve the attention of nurses. In the human need for immune regulation, diagnoses such as “Infection”, “Risk of Infection”, “Vaccination Status, Impaired” and “Colostrotherapy Regime” as statements that obtained good indexes (all with CVI = 1 in relation to relevance). Newborns admitted to hospital are a population susceptible to infections that have characteristics not seen in any other group of patients. There are numerous risk factors associated with patient characteristics, hospital stay and the level of care provided, which can increase the risk of hospital-acquired infections^(17)^.

Another point worth highlighting in immune regulation, but this time in a positive way, is colostrum therapy, where maternal colostrum is administered directly into the newborn’s oropharynx to promote a systemic effect, favoring the development of the immune and gastrointestinal systems. Interventions involving the administration of oropharyngeal colostrum to premature newborns are possible and plausible strategies in neonatal health services^(18)^.

With regard to the need for cutaneous-mucosal integrity, a need that garnered the most statements, we highlight the statements “Impaired Skin Integrity”, “Thin Skin”; “Gelatinous Skin” and “Risk of Periostomal Skin Complication” (all with CVI = 1 in relation to relevance). In fact, these statements are evident in premature newborns since there is an underdevelopment of the cutaneous-mucosal barrier. The skin of premature newborns only develops to normal levels of functionality after three weeks of life, due to less contact with the vernix caseosa and the fact that the stratum corneum is less thick, exposing the skin to a greater risk of damage^(19)^. In addition to these factors, there are problems resulting from iatrogenesis, surgeries, malformations and the constant need to use invasive devices to aid therapy.

On the other hand, it is necessary to discuss the human need for nutrition, where it is known that in the newborn, this need is often altered, since the introduction of enteral or parenteral nutrition is a delicate process. Due to the characteristics of prematurity, it is initially not possible to feed by sucking, requiring the use of gavage through the enteral tube^(20)^.

It is therefore known that in premature babies, breast milk plays an important role in their development, but mothers encounter barriers to breastfeeding. The more premature the baby, the less chance they have of being breastfed, because most mothers, although they start breastfeeding immediately, less than half continue later and this can be attributed to insufficient family support, young maternal age and less schooling^(21)^.

The need for vascular regulation is highlighted by diagnoses such as “Bradycardia”, “Tachycardia”, “Hypertension”, “Shock” and “Risk of Intracranial Hemorrhage” (all with CVI = 1 in relation to relevance). These phenomena are observed in different situations frequently experienced by this population, such as problems in the respiratory and cardiovascular systems, as well as in the response to pain and/or stressful stimuli^(22)^.

In relation to the need for thermal regulation, the diagnoses of “Risk of Hyperthermia”, “Risk of Hypothermia” and “Cyanosis of Extremities” (all with CVI = 1 in relation to relevance) are diagnoses present in the nurses’ records, as they are recurrent phenomena. A study which aimed to analyze the temperature pattern of low-birth-weight newborns admitted to a Brazilian maternity hospital showed that on admission the average axillary temperature was 34.98°C. The rate of hypothermia on admission was considerably serious, with the percentage of hypothermic newborns (<36.5°C) in the first hour, six hours and 12 hours of hospitalization being 93.33%, 73.33% and 57.78%, respectively^(23)^.

On the other hand, hyperthermia is also observed, as the heating devices that would be great alternatives for controlling hypothermia are sometimes used inappropriately, leading to undesirable results such as hyperthermia, which can cause fluid evaporation, skin dryness and can lead to insensible water loss^(24)^.

The diagnoses of “hypoglycemia” and “hyperglycemia” (all with CVI=1 in relation to relevance), related to the need for hormonal regulation, occur especially in preterm newborns, in septic conditions, as a result of an inadequate response to insulin, surgical stress, infusion of glucose or lipids through venous hydration and/or parenteral nutrition, glucose infusion rate higher than tolerated or inadequate prescription/infusion of prolonged parenteral nutrition. Hypoglycemia can occur due to low glucose reserves, the inability of the newborn to feed and easy heat loss^(17)^.

With regard to the need for oxygenation, it is essential for preterm newborns, since the use of oxygen is one of the most commonly used therapies in neonatology and respiratory distress syndrome (RDS) is one of the main causes of morbidity and mortality in this population. The lower the gestational age, the greater the chance of a premature baby needing ventilatory assistance and due to the immaturity of the lungs, mechanical ventilation is the life support treatment for these patients. Even with the most modern treatments and the reduction in premature infants requiring intubation and mechanical ventilation, a proportion of them still need ventilator support^(25)^.

Preterm infants admitted to the NICU are susceptible to changes in the need for neurological regulation due to the immaturity of the nervous system, which can manifest itself in hypoactivity, decreased reflexes and convulsions. The abnormal electrical discharge of the central nervous system has numerous etiologies, including hypoxic-ischemic lesions and malformations of the central nervous system^(26)^.

Alterations in electrolyte and hydro-saline regulation, hydration and elimination are problems related to prematurity and can be seen in the quantity of fluids eliminated and/or accumulated in the tissues and are manifested by signs such as diarrhea and dehydration. A study of 96 newborns hospitalized for 6 months identified some of the priority diagnoses in this population and the risk of electrolyte imbalance appeared in 91% of the observations^(27)^.

Related to the need to regulate cell growth, the statements listed included phenomena related to growth (cell multiplication) and development. Due to the intrinsic characteristics of prematurity, associated with the length of hospitalization and separation from parents, one study showed the nursing diagnosis “Risk of Developmental Delay” in 100% of the children observed. In addition, diagnoses linked to alterations and mismatches in the autonomous, motor, behavioral and regulatory subsystems were also noted in this study^(27)^.

As for the need for physical safety and the environment, we can say that the environment in the NICU interacts with the newborn in an extremely different way to what the premature infant experienced in the mother’s womb. It is in the NICU that complex and specialized treatments and care are developed for the survival and prevention of complications of prematurity^(27)^. However, this is not an environment free from aggressive agents and sometimes, even in those units concerned with care that is considered humanized, they need handling that offers the indicated therapy, but which may cause them some kind of harm^(28)^.

The needs for emotional security, love and gregariousness are part of the psychosocial needs and the nursing diagnoses included in them reflect the impact on the family of the hospitalization of newborns in the NICU. Premature birth is a multi-problematic event and can have a negative impact on both the mother-father relationship and parent-child interactions. A study carried out in Italy with parents of preterm newborns admitted to a NICU concluded that premature birth seems to be particularly stressful for younger mothers and fathers and that the lack of knowledge about what to do and what to expect caused greater stress. In addition, the NICU environment itself already contributes to increasing maternal stress levels^(29)^.

The statements presented here have been rigorously validated and reflect the reality of the nurses. In addition, the adoption of Horta’s theoretical model provided guidance for the statements and practical clarity for locating the nurses. The use of this model helped to bring together the theoretical concepts and the meanings attributed to the diagnoses and their interrelationship with care, making it possible to reflect on and organize their practices in accordance with the needs of their clients^(30)^. In addition, the theory is in line with the institutional philosophy, the health clientele studied and the terminology chosen.

By verifying the phenomena experienced in nursing practice, nurses are able to construct statements based on the clinical condition of the individual under their care, since nursing diagnosis is a way of expressing the care needs that occur in their practice and require clinical reasoning in relation to nursing problems. In addition, the unification of professional language and the use of terms that are more appropriate to real health needs makes it possible to evaluate care and generate indicators, contributing to the construction and implementation of care instruments, with a focus on patient safety.

The study’s limitations include the collection of data from medical records, carried out manually (without the aid of any ontology-building tool), and the availability of the judges to return the validation form. It is strongly recommended that a terminological subset should be created, and then clinically validated in order to assess its effectiveness and operationality in practice, and that new research involving the ICNP^®^ for premature newborns be carried out in order to further improve nursing care.

## CONCLUSION

It is considered that the proposed objective was achieved since 145 nursing diagnosis statements were constructed and validated, reflecting the reality of specialist nurses. Adapting to basic human needs was beneficial in terms of the theoretical direction of the statements, with a greater number of statements relating to the human need for cutaneous-mucosal integrity.

It is hoped that this material will serve as an easily accessible reference for neonatal nurses so that they can draw up individualized care plans, basing their practice on evidence, optimizing the time available and contributing to greater visibility of the nurse’s work process within the multi-professional team.

The contribution of this study reflects the valuable and delicate specificity of the area and, consequently, the strengthening of the ICNP^®^, given the possibility of new terms and statements being added to the terminology. It should be noted that the construction of a subset will complement and encourage clinical reasoning and decision-making by nurses, as they will be provided with detailed documentation and records of their practice, regardless of the scenario in which the newborn is.

Beyond the field of neonatal nursing, it can be seen that an instrument that supports the application of the nursing process, using specialized language terminology based on a theory, in turn strengthening Nursing as a science, as well as facilitating the applicability and implementation of this nursing process in care practice based on everyday phenomena.

## References

[1] Chawanpaiboon S, Vogel JP, Moller AB, Lumbiganon P, Petzold M, Hogan D (2019). Global, regional, and national estimates of levels of preterm birth in 2014: a systematic review and modelling analysis.. Lancet Glob Health..

[2] World Health Organization. (2022). WHO recommendations for care of the preterm or low birth weight infant [Internet]..

[3] Conselho Federal de Enfermagem (BR). (2009). Resolução Nº 358 do Conselho Federal de Enfermagem, de 15 de outubro de 2009 (BR) [Internet]..

[4] Garcia TR, Nóbrega MML, Cubas MR, Classificação Internacional para a Prática de Enfermagem - CIPE^®^: versão 2019. (2019). Centro de Pesquisa e Desenvolvimento da CIPE® da Universidade Federal da Paraíba [Internet]..

[5] Tommasi V, Vercesi G, Sannino P, Bassola B, Plevani L, Cilluffo S (2022). The use of International Classification for Nursing Practice (ICNP^®^) inpediatric and neonatal settings: literature review.. PROF.INF. [Internet]..

[6] Menezes HF, Camacho ACLF, Nóbrega MML, Fuly PSC, Fernandes SF, Silva RAR (2020). Paths taken by Brazilian Nursing for the development of terminological subsets.. Rev Lat Am Enfermagem..

[7] Querido DL, Christoffel MM, Almeida VS, Rodrigues EC, Lins SMSB, Jennings JM (2022). Specialized nursing terminology for premature newborns in neonatal intensive care units.. Rev. Eletr. Enferm..

[8] Prado NCC, Lima DM, Silva ABP, Mercês BMO, Menezes HF, Silva RAR (2022). Elaboration and validation of a terminology subset for newborns with central venous catheters.. Texto Contexto Enferm..

[9] Prado NCC, Menezes HFM, Sousa PAF, Lopes DCL, Santos FR, Santos RSC (2022). Terms of specialized nursing language in the care of the newborn with central venous catheter.. Rev Bras Enferm.

[10] Menezes HF, Camacho ACLF, Sousa PAF, Primo CC, Ferreira LB, Silva RAR (2021). Validation of Nursing Diagnoses for people with chronic kidney conditions on conservative treatment.. Rev Esc Enferm USP..

[11] Carvalho CMG, Cubas MR, Nóbrega MML (2017). Brazilian method for the development terminological subsets of ICNP^®^: limits and potentialities.. Rev Bras Enferm..

[12] International Organization for Standardization. (2014). ISO 18104: health informatics: categorial structures for representation of nursing diagnoses and nursing actions in terminological systems..

[13] Horta WA (2011). Processo de enfermagem.

[14] Cubas MR, Nóbrega MML (2022). Equivalence between ICNP® and SNOMED CT concepts: theoretical reflection.. Texto Contexto Enferm..

[15] Coluci MZO, Alexandre NMC, Milani D (2015). Construção de instrumentos de medida na área da saúde.. Ciênc Saúde coletiva.

[16] Neri E, Genova F, Monti F, Trombini E, Biasini A, Stella M (2020). Developmental dimensions in preterm infants during the 1st year of life: the influence of severity of prematurity and maternal generalized anxiety.. Front Psychol.

[17] Rangelova V, Kevorkyan A, Krasteva M (2020). Nosocomial infections in the neonatal intensive care unit.. Arch Balk Med Union..

[18] Martins CC, Ramos MSX, Amaral MVC, Costa JSP, Cerqueira ES, Vieira TO (2020). Colostrum oropharyngeal immunotherapy for very low birth weight preterm infants: protocol of an intervention study.. BMC Pediatr..

[19] Neonatologist S (2021). Guidelines for neonatal skin management in the neonatal intensive care unit (2021).. Chinese Journal of Contemporary Pediatrics..

[20] Viswanathan S, Jadcherla S (2020). Feeding and swallowing difficulties in neonates: developmental physiology and pathophysiology.. Clin Perinatol..

[21] Zukova S, Krumina V, Buceniece J (2021). Breastfeeding preterm born infant: chance and challenge.. Int J Pediatr Adolesc Med..

[22] Saxton SN, Walker BL, Dukhovny D (2021). Parents matter: examination of family presence in the neonatal intensive care unit.. Am J Perinatol..

[23] Aquino ARG, Silva BCO, Barreto VP, Aquino ARG, Trigueiro EV, Feijão AR (2021). Profile of risky newborns related to thermoregulation in a Neonatal Intensive Care Unit.. Enferm Glob..

[24] Wood T, Johnson M, Temples T, Bordelon C (2022). Thermoneutral environment for neonates: back to the basics.. Neonatal Netw..

[25] Yue G, Wang J, Li H, Li B, Ju R (2021). Risk factors of mechanical ventilation in premature infants during hospitalization.. Ther Clin Risk Manag..

[26] Acar DB, Bulbul A, Uslu S (2019). Current overview of neonatal convulsions.. Sisli Etfal Hastan Tip Bul..

[27] Taghinejad F, Nikfarid L, Monfared MN, Hoseini N, Habibi S (2021). Nursing diagnoses of preterm infants in the neonatal intensive care unit: a cross sectional study.. J Neonatal Nurs..

[28] Brasil, Ministério da Saúde, Secretaria de Atenção Primária à Saúde, Departamento de Ações Programáticas Estratégicas. (2018). Método canguru: diretrizes de cuidado [Internet]..

[29] Ionio C, Mascheroni E, Colombo C, Castoldi F, Lista G (2019). Stress and feelings in mothers and fathers in NICU: identifying risk factors for early interventions.. Prim Health Care Res Dev.

[30] Siega CK, Adamy EK, Toso BRGO, Zocche DAZ, Zanatta EA (2020). Lived experiences and meanings of the nurse consultation in childcare: analysis in the light of Wanda Horta.. Rev Enferm UFSM..

